# Azole-Resistant *Aspergillus fumigatus*, Iran

**DOI:** 10.3201/eid1905.130075

**Published:** 2013-05

**Authors:** Seyedmojtaba Seyedmousavi, Seyed Jamal Hashemi, Ensieh Zibafar, Jan Zoll, Mohammad T. Hedayati, Johan W. Mouton, Willem J.G. Melchers, Paul E. Verweij

**Affiliations:** Radboud University Nijmegen Medical Centre, Nijmegen, the Netherlands (S.Seyedmousavi, J.W Mouton, W.J.G.Melchers, P.E.Verweij);; Nijmegen Institute for Infection, Inflammation and Immunity, Nijmegen (S.Seyedmousavi, J.W Mouton, W.J.G.Melchers, P.E.Verweij);; Tehran University of Medical Sciences, Tehran, Iran (S.J.Hashemi, E.Zibafar);; Mazndaran University of Medical Sciences, Sari, Iran (M.T.Hedayati)

**Keywords:** azole resistance, *Aspergillus fumigatus*, TR_34_/L98H, Middle East, Iran, fungi

**To the Editor:** *Aspergillus fumigatus* causes a variety of diseases in humans. The drugs recommended for treatment of *Aspergillus* diseases are the mold-active azole antifungal drugs (*1*). However, a wide range of mutations in *A. fumigatus* confer azole resistance, which commonly involves modifications in the *cyp51A* gene (*2*), the target for azole antifungal drugs.

Azole resistance is thought to be selected for as a result of patient therapy or exposure to azole compounds in the environment; resistance in clinical *A. fumigatus* isolates has been increasingly reported in several European countries, Asia, and the United States (*3*–*7*). The most frequently reported resistance mechanism is a 34-bp tandem repeat (TR_34_) in combination with a substitution at codon 98 (TR_34_/L98H) (*4*); this mechanism is believed to have been selected for through environmental exposure to azole fungicides.

Because routine in vitro susceptibility testing of clinical *Aspergillus* isolates is not common in many centers worldwide, the prevalence of azole resistance might be underestimated. We investigated the prevalence of azole resistance in clinical *A. fumigatus* isolates stored for 6 years (2003–2009) at Tehran University Mycology Reference Centre and Islamic Azad University, Ardabil Branch, Iran.

We investigated 124 clinical *A. fumigatus* isolates obtained from patients with *Aspergillus* diseases (Technical Appendix Table 1). We conducted strain identification, in vitro antifungal susceptibility testing, and sequence-based analysis of the *Cyp51A* gene, as described (*4*). We performed microsatellite genotyping of all *A. fumigatus* isolates for which the MIC of itraconazole was ≥16 mg/L (*8*) by using a short tandem repeat *A. fumigatus* assay, and we compared the results with those reported for the Netherlands (20 isolates) and other European countries (24 isolates) (Technical Appendix Figure).

The distribution of azole-resistant and wild-type *A. fumigatus* isolates examined in this study, according to year of isolation, is shown in online Technical Appendix Table 1. Of 124 *A. fumigatus* isolates, 4 grew on the wells containing itraconazole and voriconazole, indicating a multidrug-resistant phenotype. Of these resistant isolates, 3 were from patients with chronic pulmonary aspergillosis and 1 was from a patient with allergic bronchopulmonary aspergillosis (Table).

**Table:** Characteristics of 4 azole-resistant clinical *Aspergillus fumigatus* isolates, Iran*

Isolate	Underlying disease	Previous azole exposure	34-bp tandem repeat†	Amino acid substitution in *cyp*51A gene‡				
					MIC, mg/L			
					Amphotericin B	Itraconazole	Voriconazole	Posaconazole
T-IR-AF 12	CPA	Yes	Positive	L98H	0.5	≥16	4.0	0.5
T-IR-AF 17	CPA	No	Positive	L98H	0.5	≥16	4.0	0.5
T-IR-AF 433	CPA	Yes	Negative	ND	0.5	≥16	8.0	0.5
T-IR-AF 890	ABPA	No	Positive	L98H	0.5	≥16	8.0	0.25

*CPA, chronic pulmonary aspergillosis; ND, not detected; ABPA, allergic bronchopulmonary aspergillosis. †34-bp tandem repeat in the promoter region of *CYP51A* gene. ‡The numbers indicate the position at which an amino acid change occurs. Nucleotides are numbered from the translation start codon ATG of *cyp51A*.

Sequence analysis of the *CYP51A* gene indicated the presence of TR_34_/L98H in 3 isolates and no mutations in the other isolate (Table). The first TR_34_/L98H isolate had been recovered in 2005, which is relatively early compared with reported isolations in other countries (Technical Appendix Table 2). Microsatellite typing of 6 short tandem repeat loci demonstrated identical patterns for 2 of the 3 azole-resistant isolates from Iran, but the TR_34_/L98H isolates from Iran did not cluster with those from the Netherlands and other European countries, indicating no close genetic relatedness (Technical Appendix Figure).

The TR_34_/L98H azole resistance mechanism was first described in 1998 in the Netherlands; since then, its presence in clinical and environmental *A. fumigatus* isolates in multiple European countries and recently in Asia has been increasingly reported (Technical Appendix Table 2) (*3*–*7*). In the study reported here, prevalence of azole resistance in clinical *A. fumigatus* isolates obtained from patients in Iran was 3.2%; most isolates exhibited the TR_34_/L98H resistance mechanism. The fact that the first TR_34_/L98H isolate was found relatively early, in 2005, underscores the possibility that prevalence of azole resistance might be underestimated in many countries because in vitro susceptibility testing of *A. fumigatus* is not routinely performed.

Microsatellite genotypic analysis of *A. fumigatus* isolates from the Netherlands and various European countries showed that the genetic diversity of TR_34_/L98H isolates is lower than that of wild-type controls (*8*). It has been suggested that TR_34_/L98H isolates might have a common ancestor that developed locally and subsequently migrated across Europe. In contrast, genotyping of TR_34_/L98H originating from India suggested a different dynamic; all environmental and clinical TR_34_/L98H isolates from India shared the same multilocus microsatellite genotype not found in any other analyzed samples, from within India or from the Netherlands, France, Germany, or the People’s Republic of China (*9*). The molecular epidemiology of the TR_34_/L98H isolates from Iran suggests that they cluster apart from the European isolates, indicating that migration from Europe to Iran, or vice versa, is unlikely. Genotyping of more TR_34_/L98H isolates from the Middle East and comparison with those from India would enhance understanding of the origin and geographic spread of TR_34_/L98H.

Our study indicates that TR_34_/L98H was in Iran in 2005; this finding adds to the growing list of regions where acquired resistance in *A. fumigatus* of environmental origin is documented. From a global perspective, fungicide use is second highest in the Asia–Pacific regions (24%), preceded only by western Europe (37%) (*10*). For a bettering understand of the scale of this emerging public health problem and for insight into the dynamics of geographic migration, surveys of fungal culture collections for TR_34_/L98H and molecular typing studies are warranted. These data would be useful not only for clinical management of *Aspergillus* diseases but also for enabling policy makers to develop strategies that prevent resistance selection by the environmental route.

Technical Appendix Distribution of azole-resistant and azole-susceptible *Aspergillus fumigatus* isolates, Iran, 2003–2009; first reports of multiple-triazole-resistant *A. fumigatus* isolate(s) carrying the TR_34_/L98H mutations in the *CYP51A* gene, by country; and minimum spanning tree comparing genotypic relatedness of clinical azole-resistant *A. fumigatus* isolates carrying TR_34_/L98H alteration in the *CYP51A* gene from Iran with those reported from European countries. TR, tandem repeat.
