# Supplementary material for: Azole-Resistant Aspergillus fumigatus, Iran
Source: Emerg Infect Dis. 2013 May;19(5):832–4. doi: 10.3201/eid1905.130075 (PMC3647519; doi:10.3201/eid1905.130075)
Supplement: Technical Appendix — Distribution of azole-resistant and azole-susceptible Aspergillus fumigatus isolates, Iran, 2003–2009; first reports of multiple-triazole-resistant A. fumigatus isolate(s) carrying the TR34/L98H mutations in the CYP51A gene, by country; and minimum spanning tree comparing genotypic relatedness of clinical azole-resistant A. fumigatus isolates carrying TR34/L98H alteration in the CYP51A gene from Iran with those reported from European countries. TR, tandem repeat. [file 13-0075-Techapp-s1.pdf]

Article DOI: http://dx.doi.org/10.3201/eid1905.130075

# Azole-resistant *Aspergillus fumigatus*, Iran

## Technical Appendix

Technical Appendix Table 1. Distribution of azole-resistant and azole-susceptible *Aspergillus fumigatus* isolates, Iran, 2003–2009

| Year of isolation | No. isolates with each phenotype and resistance mechanism | | |
|---|---|---|---|
| | Wild type | Resistant, $TR_{34}$/L98H | Resistant, non-$TR_{34}$/L98H |
| 2003 | 11 | – | – |
| 2004 | 18 | – | – |
| 2005 | 11 | 1 | – |
| 2006 | 17 | – | – |
| 2007 | 19 | 2 | 1 |
| 2008 | 20 | – | – |
| 2009 | 24 | – | – |
| Total | 120 | 3 | 1 |

Technical Appendix Table 2. First reports of multiple-triazole-resistant *Aspergillus fumigatus* isolate(s) carrying the $TR_{34}$/L98H mutations in the *CYP*51A gene, by country

| Region/Country | | First reported $TR_{34}$/L98H *A.fumigatus* isolate(s) | Reference |
|---|---|---|---|
| **Europe** | Netherlands | 1998 | Snelders et al. 2008 (*1*) |
| | UK | 1999 | Howard et al. 2009 (*2*) |
| | Norway | 2001 | Snelders et al. 2008 (*1*) |
| | Spain | 2002/2003 | Mellado et al. 2012 (*3*) |
| | Denmark | 2007 | Mortensen et al. 2011 (*4*) |
| | Belgium | 2008 | Lagrou et al. 2008 (*5*) |
| | France | 2010/2011 | Burgel et al. 2012 (*6*), Morio et al. 2012 (*7*) |
| | Germany | 2012 | Rath et al. 2012 (*8*) |
| **Asia** | Iran | 2005 | Present study |
| | India | 2008 | Chowdhary et al. 2012 (*9*,*10*) |
| | China | 2008/2009 | Lockhart et al. 2011 (*11*) |

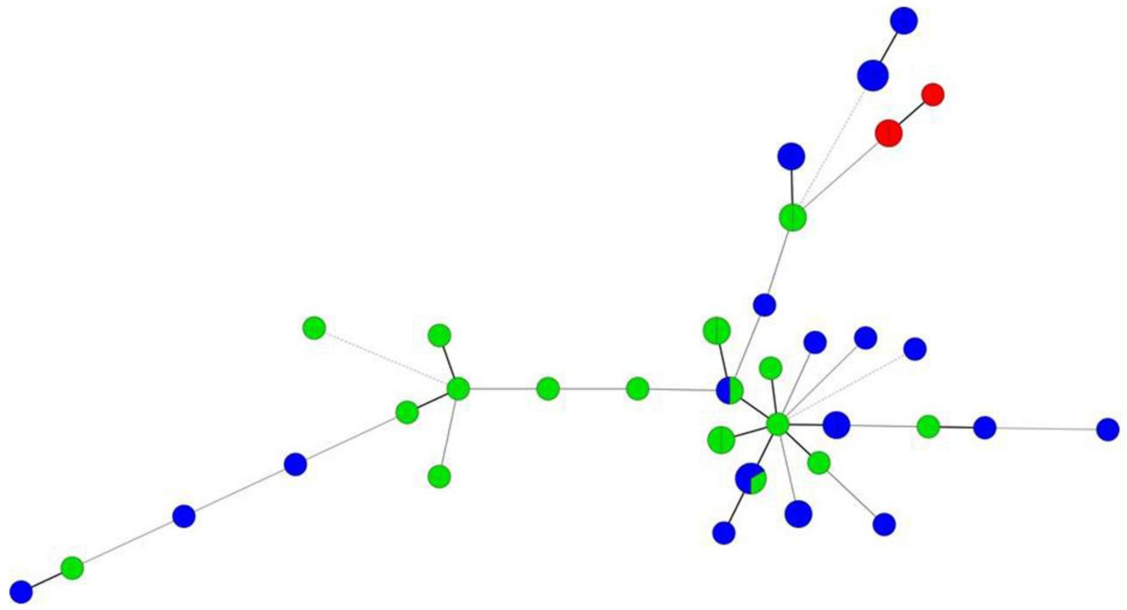

Technical Appendix Figure. Minimum spanning tree comparing genotypic relatedness of clinical azole-resistant *Aspergillus fumigatus* isolates carrying $TR_{34}$/L98H alteration in the *CYP*51A gene from Iran with those reported from European countries. Microsatellite typing of 6 STR loci demonstrated identical patterns for two of the three azole-resistant isolates from Iran, but the $TR_{34}$/L98H isolates from Iran did not cluster with those from the Netherlands and other European countries, indicating no close genetic relatedness. Each circle corresponds to a unique genotype, and each color indicates the origin of azole-resistant $TR_{34}$/L98H isolates: red, Iran (n = 3); green, the Netherlands (n = 20); blue, other European countries (n = 24). The size of the circle corresponds to the number of isolates with that genotype. Connecting lines correspond to the number of different microsatellite loci between the genotypes: solid thick and thin branches indicate 1 and 2 microsatellite marker differences, respectively; dashed branches indicate 3 microsatellite marker difference; dotted branches indicate $\geq$4 microsatellite marker differences between genotypes.

**References**

1. Snelders E, van der Lee HA, Kuijpers J, Rijs AJ, Varga J, Samson RA, et al. Emergence of azole resistance in *Aspergillus fumigatus* and spread of a single resistance mechanism. PLoS Med. 2008;5:e219. PubMed http://dx.doi.org/10.1371/journal.pmed.0050219

2. Howard SJ, Cerar D, Anderson MJ, Albarrag A, Fisher MC, Pasqualotto AC, et al. Frequency and evolution of azole resistance in *Aspergillus fumigatus* associated with treatment failure. Emerg Infect Dis. 2009;15:1068–76. PubMed http://dx.doi.org/10.3201/eid1507.090043

3. Mellado E, De La Camara R, Buendia B, Rodriguez-Tudela JL, Cuenca-Estrella M. Breakthrough pulmonary *Aspergillus fumigatus* infection with multiple triazole resistance in a Spanish patient with chronic myeloid leukemia. Rev Iberoam Micol. 2013;30:64–8. PubMed http://dx.doi.org/10.1016/j.riam.2012.09.002

4. Mortensen KL, Jensen RH, Johansen HK, Skov M, Pressler T, Howard SJ, et al. *Aspergillus* species and other molds in respiratory samples from patients with cystic fibrosis: a laboratory-based study with focus on Aspergillus fumigatus azole resistance. J Clin Microbiol. 2011;49:2243–51. PubMed http://dx.doi.org/10.1128/JCM.00213-11

5. Lagrou K, Vleeschouwer J, Meersseman W, Dupont L, Verleden GM, Melchers WJG, et al., editors. Triazole resistance among clinical *Aspergillus fumigatus* isolates. 3rd Adv Against Aspergillosis. 2008; 16–19 January 2008; Miami, FL.

6. Burgel PR, Baixench MT, Amsellem M, Audureau E, Chapron J, Kanaan R, et al. High prevalence of azole-resistant *Aspergillus fumigatus* in adults with cystic fibrosis exposed to itraconazole. Antimicrob Agents Chemother. 2012;56:869–74. PubMed http://dx.doi.org/10.1128/AAC.05077-11

7. Morio F, Aubin GG, Danner-Boucher I, Haloun A, Sacchetto E, Garcia-Hermoso D, et al. High prevalence of triazole resistance in *Aspergillus fumigatus*, especially mediated by TR/L98H, in a French cohort of patients with cystic fibrosis. J Antimicrob Chemother. 2012;67:1870–3. PubMed http://dx.doi.org/10.1093/jac/dks160

8. Rath PM, Buchheidt D, Spiess B, Arfanis E, Buer J, Steinmann J. First reported case of azole-resistant *Aspergillus fumigatus* due to the TR/L98H mutation in Germany. Antimicrob Agents Chemother. 2012;56:6060–1. PubMed http://dx.doi.org/10.1128/AAC.01017-12

9. Chowdhary A, Kathuria S, Xu J, Sharma C, Sundar G, Singh PK, et al. Clonal expansion and Emergence of eEnvironmental multiple-triazole-resistant *Aspergillus fumigatus* strains carrying the TR(34)/L98H mutations in the *cyp51A* gene in India. PLoS ONE. 2012;7:e52871. PubMed http://dx.doi.org/10.1371/journal.pone.0052871

10. Chowdhary A, Kathuria S, Randhawa HS, Gaur SN, Klaassen CH, Meis JF. Isolation of multiple-triazole-resistant *Aspergillus fumigatus* strains carrying the TR/L98H mutations in the *cyp51A* gene in India. J Antimicrob Chemother. 2012;67:362–6. PubMed http://dx.doi.org/10.1093/jac/dkr443

11. Lockhart SR, Frade JP, Etienne KA, Pfaller MA, Diekema DJ, Balajee SA. Azole resistance in *Aspergillus fumigatus* isolates from the ARTEMIS global surveillance study is primarily due to the TR/L98H mutation in the cyp51A gene. Antimicrob Agents Chemother. 2011;55:4465–8. PubMed http://dx.doi.org/10.1128/AAC.00185-11
